# Psychological and somatic distress in Chinese outpatients at general hospitals: a cross-sectional study

**DOI:** 10.1186/s12991-017-0158-y

**Published:** 2017-10-16

**Authors:** Nana Xiong, Jing Wei, Kurt Fritzsche, Rainer Leonhart, Xia Hong, Tao Li, Jing Jiang, Liming Zhu, Guoqing Tian, Xudong Zhao, Lan Zhang, Rainer Schaefert

**Affiliations:** 1 Department of Psychological Medicine, Peking Union Medical College Hospital, Chinese Academy of Medical Sciences & Peking Union Medical College, Beijing, 100730 People’s Republic of China; 2 Department of Psychosomatic Medicine and Psychotherapy, University Medical Centre Freiburg, Freiburg, Germany; 3 Institute of Psychology, University of Freiburg, Freiburg, Germany; 4 Department of Gastroenterology, Peking Union Medical College Hospital, Chinese Academy of Medical Sciences & Peking Union Medical College, Beijing, China; 5 Department of Traditional Chinese Medicine, Peking Union Medical College Hospital, Chinese Academy of Medical Sciences & Peking Union Medical College, Beijing, China; 6 Department of Psychosomatic Medicine, Dongfang Hospital, School of Medicine, Tongji University, Shanghai, China; 7 Mental Health Centre, West China Hospital, Sichuan University, Chengdu, Sichuan China; 8 Department of General Internal Medicine and Psychosomatics, University Hospital Heidelberg, Heidelberg, Germany

**Keywords:** China, Discriminant analysis, General hospital outpatients, Psychological distress, Somatic distress

## Abstract

**Background:**

Our study aimed (1) to describe the proportion of psychological distress among Chinese outpatients at general hospitals, (2) to compare cognitive and behavioral characteristics of patients with different distress patterns, and (3) to investigate the discriminant function of the analyzed variables in indicating the affinity towards the different distress patterns.

**Methods:**

This multicenter cross-sectional study was conducted at ten outpatient departments at Chinese general hospitals. The somatic symptom severity scale (PHQ-15), the nine-item depression scale (PHQ-9), and the seven-item anxiety scale (GAD-7) were employed to classify patients in terms of four distress patterns.

**Results:**

A total of 491 patients were enrolled. Among them, the proportion of patients with high psychological distress was significantly higher within those with high somatic distress (74.5% vs. 25.5%, *p* < .001). Patients with psychological distress alone and mixed distress were significantly younger and with lower monthly family income, while the proportion of female patients (80.9%) was highest in the somatic distress group. Patients with mixed distress had the most negative cognitive and behavioral characteristics [highest health anxiety (5.0 ± 1.9), lowest sense of coherence (35.5 ± 10.0), the worst doctor–patient relationship from both patients’ (36.0 ± 7.3) and doctors’ perspectives (23.3 ± 7.0)], as well as most impaired quality of life (41.6 ± 7.4 and 31.9 ± 10.3). In addition, compared with patients with somatic distress alone, those with psychological distress alone had lower sense of coherence, worse doctor–patient relationship, and more impaired mental quality of life, but less doctor visits. Discriminant analysis showed that gender, mental quality of life, health anxiety, sense of coherence, and frequent doctor visits were significant indicators in identifying patients with different distress patterns.

**Conclusions:**

Our study found that (1) psychological distress was not rare in the Chinese general hospital outpatients, especially in those with high somatic distress; (2) patients with psychological distress alone sought less help from doctors, despite their severe psychosocial impairment; and (3) gender, health anxiety, sense of coherence, mental quality of life, and frequent doctor visits could help to identify different distress patterns.

## Background

The most common mental disorders worldwide are depression, anxiety, and somatoform disorders [1–6]. A recent population-based survey on the most frequent mental disorders in Europe found the following prevalence rates: 14.0% for anxiety disorders, 6.9% for major depression, and 6.3% for somatoform disorders [7]. Among those disorders, high comorbidity rates have been observed, leading to further impairment in health-related quality of life (QoL) [8–11].

However, the prevalence of depression, anxiety, and somatoform disorders in China differed greatly under different research backgrounds. For example, the lifetime prevalence of affective disorders was only reported as 0.08% in 1998 [12]. Therefore, previously, it was believed that Chinese people are more likely to express somatic symptoms, rather than emotional distress [13, 14]. Nevertheless, more recent epidemiological studies in China have suggested that the rates of depression and anxiety disorders were comparable with those reported in Western countries [15, 16]. A recent study conducted in the Hong Kong general population detected both common somatic and psychological distress [17]. Just like the dimensions of stability and extroversion used to describe the personality types, patients were classified into four distress patterns according to their scores on both the somatic and the psychological dimensions. Even though such categorization was not as rigorous as psychiatric diagnoses, it provided a simple way to assess and distinguish clusters of symptoms among a large sample. Besides, previous research suggested that the elevated self-rated was consistent with the diagnoses of depression/anxiety/somatoform disorders by the general practitioners [1]. Therefore, inspired by the above results, we intend to clarify the proportion of psychological and somatic distress among mainland Chinese general hospital outpatients and the associated frequency of doctor visits.

In addition, a deterioration of the doctor–patient relationship (DPR) has emerged as a highly visible risk in China. As commented by the Lancet, “a third of doctors have experienced conflict and thousands have been injured; the scale, frequency, and viciousness of attacks have shocked the world” [18, 19]. Besides health-care reform and improving hospital governance, to solve the problem of patients’ dissatisfaction and violence against doctors, trustful DPR needs to be rebuilt bottom–up. Therefore, it is essential to first to understand what happens in the real world and how patients with different distress patterns experience their DPR. Previous studies found that somatizing patients were less satisfied with their doctors [20], especially when they believed that they were not being understood or did not obtain clear diagnoses or explanations [21]. On the other hand, doctors also experienced more difficulties with patients with multiple somatic symptoms and comorbid mental disorders [22, 23]. However, whether the DPR differed among patients with different distress patterns remained unclear.

Furthermore, how patients understand their symptoms or illness is essential, as it influences their coping behavior [24, 25] and consecutive health outcomes [26]. In our study, we used health anxiety and sense of coherence (SOC) as key elements to reflect illness-related cognitions. Health anxiety has been found to be closely associated with somatization and hypochondriasis in both Western [27] and Chinese populations [28]. The SOC is a theoretical framework that provides an explanation of the role of distress in human functioning, considering comprehensibility, manageability, and meaningfulness when confronted with illnesses [29]. Individuals with strong SOC are capable of perceiving stressors rationally and remain healthy when facing stressful events. Previous studies conducted in China showed that lower SOC were associated with higher depression levels among patients with post-stroke depression [30], and that the SOC of patients with acquired immune deficiency syndrome (AIDS) was lower than the national norms [31]. However, studies were conducted among separate groups of patients with physical or mental illnesses, while the similarities and differences of the cognitive characteristics of patients with different distress patterns remained unclear.

Our previous research has showed that patients with high somatic distress were associated with psycho-behavioral features like “catastrophising” and “illness vulnerability” [32]. Comparing with it, this secondary data analysis shifted the focus to the level of psychological distress, and its combination with different level of somatic distress. Besides, unlike the previous research, general health-related indicators were employed for comparisons, like the sense of coherence, and the doctor–patient relationship. Therefore, the aims of this research were threefold: (1) to describe the proportion of psychological distress among Chinese general hospital outpatients; (2) to compare the cognitive and behavioral characteristics patients with different distress patterns; and (3) to investigate the discriminant function of the external sociodemographic, cognitive and behavioral variables in indicating the affinity towards the different distress patterns.

## Methods

### Study design and setting

A detailed enrollment procedure has already been published [32]. The data was collected in a multicenter cross-sectional study conducted between February 1, 2011 and October 30, 2012 at 10 general hospital outpatient clinics in Beijing, Shanghai, Chengdu, and Kunming. The neurology and gastroenterology departments were chosen to represent the modern biomedicine model. Traditional Chinese Medicine (TCM) departments were selected to represent the traditional medicine model. Psychological medicine departments were chosen to represent the psychosomatic medicine model. On randomly selected screening days, all consecutive patients who entered one of the participating departments were informed about the study and invited to participate by research assistants. All participants were screened using the 15-item patient health questionnaire (PHQ-15) to assess the severity of somatic symptoms. Recruitment continued until a sample size of 25 patients with high and low somatic distress was enrolled in each medical setting.

### Subjects

The inclusion criteria for the study were as follows: 18 years or older, seeking treatment voluntarily for their own problems, and able to read and sign the informed consent form. The exclusion criteria included language barriers or limited writing skills in Mandarin Chinese, cognitive impairment/organic brain disorder/dementia, psychosis, and acute suicidal tendency. The named criteria were clinically assessed by both research assistants (medical students) and medical doctors.

Written informed consent was obtained from all eligible participants. For data analysis, all questionnaires were copied and sent to the study center located at the medical center of Freiburg University, where all data were entered, stored and monitored. The study was approved by the ethics committees of the two principal investigators’ (XZ and KF) universities as well as the Shanghai Dong Fang Hospital and the University Medical Centre Freiburg.

### Assessment instruments

#### The 15-item patient health questionnaire (PHQ-15)

The PHQ-15 includes 15 prevalent somatic symptoms or symptom clusters that represent over 90% of the symptoms observed in primary care [33]. Studies in both Western and Chinese populations have exhibited the satisfactory reliability and validity of the PHQ-15 [33–36]. The cut-off score of 10 points was adopted to separate patients with high or low somatic distress, since it was previously identified as optimal for predicting the diagnosis of somatoform disorder [37]. The Cronbach’s alpha of the PHQ-15 was .80 in this study. An additional question was included about the symptom duration, and the responses were divided into five categories (“fewer than 4 weeks”, “4 weeks to 6 months”, “6 months to 1 year”, “1 to 2 years” and “greater than 2 years”).

#### The nine-item patient health questionnaire (PHQ-9)

It was used to measure the severity of depression. Respondents were asked to rate the frequency of the symptoms indicated during the past 2 weeks between 0 (not at all) and 3 (nearly every day), resulting in a total score ranging from 0 to 27. It was proved to be reliable and valid to detect major depression in Chinese patients with multiple somatic symptoms at the cut-off point of 10 (sensitivity = .77, specificity = .76) [38]. The Cronbach’s alpha of the PHQ-9 was .89 in this study.

#### The seven-item anxiety scale (GAD-7)

The questionnaire was used to measure the severity of generalized anxiety. A meta-analysis suggested that the GAD-7 had good operating characteristics for detecting generalized anxiety, panic, social anxiety and post-traumatic stress disorder with an optimal cut-off point of 10. Using this cut-off point, the GAD-7 has demonstrated good reliability and validity in screening anxiety disorders in Chinese general hospital outpatients [39]. The Cronbach’s alpha of the GAD-7 was .92 in this study.

#### The Whiteley-7 scale (WI-7)

The instrument was used to assess health-related anxiety by seven items, such as “do you think there is something seriously wrong with your body”. It has demonstrated good sensitivity and specificity for screening DSM-IV somatization disorder and hypochondriasis in primary care samples [40, 41]. The Chinese version of the WI-7 has exhibited satisfactory reliability and internal validity in the general population [28]. The Cronbach’s alpha of the WI-7 was .75 in this study.

#### The nine-item sense of coherence scale (SOC-9)

It was employed to assess meaningfulness, comprehensibility and manageability when confronted with illnesses, by asking “do you have the feeling that you are in an unfamiliar situation and don’t know what to do”, etc. Since the factorial validity of the original SOC-29 was found to be problematic [42], this brief scale was recommended to provide a single-factor solution, with higher scores reflecting a stronger SOC [43]. Our previous research showed that the Chinese version of the SOC-9 was reliable with Chinese general outpatients [44]. The Cronbach’s alpha of the SOC-9 was .82 in this study.

#### The patient–doctor relationship questionnaire (PDRQ-9) and the difficult doctor–patient relationship questionnaire (DDPRQ-10)

They were employed to measure the DPR from patients’ and doctors’ perspectives, respectively. The PDRQ-9 was derived from the Helping Alliance Questionnaire [45]. Nine items, such as “my doctor helps me”, are rated on a Likert-scale from 1 (not appropriate at all) to 5 (totally appropriate), with higher sum scores indicating a better DPR. The doctor-rated DDPRQ-10 was used to measure how difficult the doctor perceived the interaction to be when caring for patients, with items like “how frustrating do you find this patient”. Higher sum scores indicate a poorer DPR (range 10–60) [22]. The Cronbach’s alpha of the PDRQ-9 and DDPRQ-10 in this study was .93 and .84, respectively. The frequency of doctor visits in the past 12 months was also assessed, and responses were divided into five categories (“0”, “1–2 times”, “3–10 times”, “11–20 times”, and “more than 20 times”).

#### The 12-item short-form health survey (SF-12)

This short version of SF-36 captures practical, reliable, and valid information on health-related QoL in the previous 4 weeks [46], which produces a physical composite score (PCS) and a mental composite score (MCS). The SF-12 has been demonstrated to be reliable and valid for use with the Chinese population [47]. The Cronbach’s alpha of the SF-12 was .78 in this study.

Since the SOC-9, PDRQ-9, and DDPRQ-10 were not yet available in Mandarin Chinese, they were translated and back-translated from German using a state-of-the-art test translation procedure. Following the “ITC-Test Adaptation Guidelines” (Version 2000) of the International Test Commission [48], independent translations were translated by three Chinese native speakers (one psychiatrist, one psychologist, and one an educator), who resided in Germany and were fluent in written and spoken German. Translations were discussed during the project meetings until the agreed versions were reached at for the next step. Then, they were back-translated into German and compared with the original German versions to create the final results [32].

### Operationalization of the somatic and psychological distress patterns

In our study, a high level of somatic distress was defined as a PHQ-15 total score ≥ 10. High psychological distress was defined as either a PHQ-9 or a GAD-7 total score ≥ 10. Thus, similar to the study performed in Hong Kong [17], our participants were divided into four groups: (1) a low-distress group with low levels of both somatic and psychological distress; (2) a somatically distressed group with a high level of somatic and a low level of psychological distress; (3) a psychologically distressed group with a low level of somatic and a high level of psychological distress; and (4) a mixed distress group with high levels of both somatic and psychological distress.

### Statistical procedures

Continuous data were presented as the means and standard deviations and compared using one-way analysis of variance (ANOVA) for the four independent groups. Analysis of covariance was employed to control for the potential bias introduced by different sociodemographic characteristics [49]. Categorical variables were described as absolute and relative frequencies and compared using Chi-square tests. Rank scaled variables were compared using the Kruskal–Wallis test. Since 12 of the 491 (2.4%) participants had missing values on the PHQ-9 and GAD-7 scales, they were replaced with the mean value of the remaining items. A *p* value of less than .05 (two-tailed) was considered to be significant.

Cramer’s *V* coefficients were calculated to reflect the associations between the four distress groups and other independent variables. Discriminant analyses were employed to investigate the discriminant function of those variables in predicting the different patterns of distress. Even though the distress patterns can be judged by the PHQ-15, PHQ-9, and GAD-7 scores, the multivariate analysis can show if there are predictors beyond these determining variables for the groups. For one thing, it can provide external evidence for the validity of the grouping. For another, it can control the confounding factors during the univariate analyses. The Wilks’ lambda stepwise method was adopted. A variable was entered into the model if its *F* value was greater than 3.84 (*p* value less than .05) and was removed if the *F* value was less than 2.71 (*p* value higher than .10) [50]. Statistical analyses were performed with IBM SPSS Statistics 20.0 and SAS 9.2.

## Results

### Study sample

Participants were recruited from the biomedical (139/243, 57.2%), TCM (148/250, 59.2%) and psychological (204/306, 66.7%) medical settings. The main reasons for not enrolling were lack of time (*n* = 137, 17.1%), lack of interest in the study (*n* = 81, 10.2%), or other reasons (*n* = 38, 4.8%), such as bad health status, lack of trust, and patients picked up prescriptions for others. Most participants were middle-aged (44.9 ± 16.4), were female (65.4%), were married (61.8%), had medical insurance (84.7%), lived in an urban area (79.6%), lived with others (87.6%), and had an education level higher than middle school (66.5%).

### Psychological distress of Chinese general hospital outpatients with high and low somatic distress

According to the study design, an equal number of participants with high and low somatic distress were recruited. Therefore, 238 (48.5%) of all respondents in our study had a PHQ-15 score ≥ 10, among whom 74.5% (149/238) also had high psychological distress. The proportion of high psychological distress in patients with low somatic distress was significantly lower [25.5% (51/253), Chi square = 91.5, *p* < .001]. Altogether, 41.1% (*n* = 202) of the patients in our sample had low distress, 18.1% (*n* = 89) had somatic distress alone, 10.4% (*n* = 51) had psychological distress alone, and 30.3% (*n* = 149) had mixed distress.

### Sociodemographic characteristics of patients with different distress patterns

As shown in Table 1, patients in both the psychologically distressed group and the mixed distress group were significantly younger. The proportion of female participants was highest in the somatic distress group. After adjustment for age and gender, the four distress groups did not differ significantly on the other sociodemographic characteristics, except that the proportion of families with a low monthly income was higher in the psychologically distressed and mixed distress groups.

**Table 1:** Sociodemographic characteristics of patients with different distress patterns (*n* = 491)

	Low-distress group (*n* = 202)	Somatically distressed group (*n* = 89)	Psychologically distressed group (*n* = 51)	Mixed distress group (*n* = 149)	*F*/*χ* ^2^ value	*p* value
Age (M ± SD)	46.5 ± 16.7^a^	47.7 ± 15.4^a^	41.2 ± 16.5^b^	42.4 ± 16.0^b^	3.6	*.014*
Female (%)	59.4	80.9	51.0	69.1	18.3	*<* *.001*
Insurance (yes %)	85.8	91.0	70.8	84.0	2.5	.060
Residence (%)					1.5	.219
City	82.7	84.3	78.4	73.0		
Rural	17.3	15.7	21.6	27.0		
Marital status (%)					1.0	.388
Single	18.0	11.2	26.0	25.9		
Married	64.5	68.5	48.0	58.5		
Divorced/widowed	17.5	20.2	26.0	15.6		
Life situation (%)					.8	.479
Alone	9.5	11.4	17.6	15.2		
With others	90.5	88.6	82.4	84.8		
Monthly family income (%)					3.1	*.027*
Less than 4000 RMB	35.6^b^	36.4^b^	52.9^a^	47.6^a^		
4000–8000 RMB	37.6	37.5	27.5	34.5		
More than 8000 RMB	26.7	26.1	19.6	17.9		
Occupation (%)					.3	.835
Employed/student	39.0	33.0	43.1	41.9		
Unemployed	37.4	38.6	27.5	29.7		
Retired	23.6	28.4	29.4	28.4		
Education (%)					2.0	.115
Elementary	10.0	13.5	6.0	11.8		
College preparatory	46.5	48.3	48.0	54.2		
University or higher	43.5	38.2	46.0	34.0		
Illness duration (%)					4.4	*.004*
< 4 weeks	22.3	9.0	11.8	5.4		
4 weeks–6 months	13.9	19.1	19.6	20.8		
6 months–1 year	14.9	13.5	7.8	12.8		
1–2 years	19.8	15.7	21.6	18.8		
> 2 years	29.2^b^	42.7	39.2^a^	42.3^a^		

Values with ^a^ were significantly higher than values with ^b^ in multi-group comparisons; age and gender were controlled for comparisons of other sociodemographic characteristics among the four distress groups

Italic values indicate significance of p value (p<0.05)

In terms of illness duration, approximately 60% of somatically/psychologically distressed/mixed distress participants had been ill for at least 1 year. Among them, the illness duration of patients with mixed distress and somatic distress was significantly longer than those with low distress.

### Cognitive characteristics of patients with different distress patterns

Cognitive characteristics were evaluated in terms of health anxiety and SOC. As measured by the WI-7 (see Table 2), mixed distressed patients had the severest health anxiety, whereas somatically and psychologically distressed patients had comparably moderate levels of health anxiety. As reflected by the salutogenic concept of SOC, patients with psychological or mixed distress seemed to manage their health significantly worse than those with low distress and somatic distress.

**Table 2:** Clinical characteristics, health-seeking behaviors, and health-related quality of life of patients with different distress patterns (*n* = 491)

	Low-distress group (*n* = 202)	Somatically distressed group (*n* = 89)	Psychologically distressed group (*n* = 51)	Mixed distress group (*n* = 149)	*F*/*χ* ^2^ value	*p* value
PHQ-15 total score	4.5 ± 2.6^d^	12.3 ± 2.3^b^	6.2 ± 2.3^c^	14.6 ± 5.3^a^	415.1	*< .001*
PHQ-9 total score	4.1 ± 2.8^c^	5.7 ± 2.4^b^	15.0 ± 4.8^a^	15.7 ± 5.2^a^	320.3	*< .001*
GAD-7 total score	2.7 ± 1.7^b^	3.7 ± 2.8^b^	11.3 ± 3.9^a^	10.9 ± 4.6^a^	155.6	*< .001*
WI-7 total score	2.6 ± 1.8^c^	4.0 ± 2.0^b^	4.0 ± 2.0^b^	5.0 ± 1.9^a^	44.2	*< .001*
SOC-9 total score	47.0 ± 8.1^a^	46.7 ± 9.1^a^	36.8 ± 10.2^b^	35.5 ± 10.0^b^	54.3	*< .001*
Doctor visits (%)					4.3	*.005*
0–2 times	35.6	27.0	37.3	25.5		
3–10 times	40.1	38.2	47.1	36.9		
11–20 times	9.4	10.1	5.9	18.1		
> 20 times	14.9	24.7	9.8	19.5		
PDRQ-9 total score	38.8 ± 5.7^a^	38.8 ± 5.7^a^	35.1 ± 7.2^b^	36.0 ± 7.3^b^	6.8	*< .001*
DDPRQ-10 total score	20.2 ± 7.3^b^	19.7 ± 6.9^b^	21.8 ± 6.4^a^	23.3 ± 7.0^a^	6.2	*< .001*
SF-12 PCS	46.4 ± 7.6^a^	44.6 ± 6.7^a^	44.7 ± 8.3^a^	41.6 ± 7.4^b^	13.7	*< .001*
SF-12 MCS	47.3 ± 8.9^a^	46.3 ± 8.6^a^	33.8 ± 8.7^b^	31.9 ± 10.3^b^	90.4	*< .001*

Values with ^a^ were significant higher than values with ^b^, values with ^b^ were significant higher than values with ^c^, and values with ^c^ were significant higher than values with ^d^ in multi-group comparison. *F*/*χ* ^2^ values and *p* values were controlled for age and gender

Italic values indicate significance of p value (p<0.05)

### Help-seeking behavioral characteristics of patients with different distress patterns

Help-seeking behaviors were measured in terms of doctor-visiting frequency and the doctor–patient relationship. As summarized in Table 2, the doctor-visiting frequency of psychologically distressed patients was similar to or even lower than that of patients with low distress. Only 15.7% of them had visited the doctor more than ten times in the past 12 months. However, approximately 35% of the participants in the somatically distressed and mixed distress groups had visited a doctor more than ten times, and approximately 20% of them had done so more than 20 times in the past year. Multi-group comparisons showed that patients with mixed or somatic distress had visited doctors significantly more frequently in the past 12 months than those with low or psychological distress.

Regarding the doctor–patient relationship, psychologically distressed and mixed distress patients rated their DPR as significantly worse than patients with low distress or somatic distress. Interestingly, doctors reported the exact same trend. That is, psychologically distressed and mixed distress patients were considered to be significantly more difficult than their counterparts with low or somatic distress.

### Health-related QoL of patients with different distress patterns

As measured by the SF-12 (see Table 2), patients with mixed distress were found to have the lowest PCS, while the other three groups of patients had similar PCS. Regarding the mental QoL, psychologically distressed and mixed distress patients were significantly more impaired than patients with low distress or somatic distress.

### Associations between the distress patterns with sociodemographic, cognitive, and help-seeking behavioral characteristics and QoL

Correlation analyses showed that the four distress groups were significantly correlated with sociodemographic [including gender (*r* = .193, *p* < .001) and health insurance (*r* = .145, *p* = .018)], illness duration (*r* = .144, *p* = .002), cognitive characteristics [including total scores of WI-7 (*r* = .319, *p* < .001), and SOC-9 (*r* = − .427, *p* < .001)], and behavioral characteristics [doctor-visiting frequency (*r* = .111, *p* = .032) and patient-experienced good DPR (*r* = .285, *p* = .031)], as well as the physical (*r* = .247, *p* < .001) and mental QoL (*r* = .582, *p* < .001).

A subsequent discriminant analysis was conducted to examine how well these external variables could help to distinguish among the four distress groups. Finally, five independent variables, including gender, mental QoL, total scores of the WI-7 and the SOC-9, and frequent doctor visits remained in the model (see Table 3), resulting in three discriminant functions. However, the third function could be ignored, since it contributed little to the model (explaining only .7% of the variance).

**Table 3:** Stepwise discriminant function analysis of patients with different distress patterns (*n* = 491)

	Unstandardized coefficient			Standardized coefficient		
	Function 1	Function 2	Function 3	Function 1	Function 2	Function 3
Gender	− .23	1.13	.11	− .11	.53	.05
SF-12 MCS	.07	.04	− .01	.65	.37	− .08
WI-7	− .20	.37	− .27	− .37	.69	− .50
SOC-9	.03	.02	− .02	.31	.18	− .19
Frequent doctor visits	− .25	.79	1.94	− .11	.36	.87
Constant	− 3.34	− 4.86	1.60	–	–	–
Variance explained	85.8%	13.5%	.7%			

As shown in Table 4, our model could correctly predict 60.7% membership of the distress patterns. However, due to the smaller group sizes of somatically distressed and psychologically distressed, there were high risks that somatically distressed participants were misclassified as with low distress (51.7%), and psychologically distressed participants were wrongly predicted as with mixed distress (64.7%).

**Table 4:** Classification results of the discriminant functions (*n* = 491)

	Predicted group membership			
	Low distress	Somatically distressed	Psychologically distressed	Mixed distressed
Low distress (*n* = 202)	(167) 82.7%	(14) 6.9%	(1) .5%	(20) 9.9%
Somatically distressed (*n* = 89)	(46) 51.7%	(19) 21.3%	(1) 1.1%	(23) 25.8%
Psychologically distressed (*n* = 51)	(17) 33.3%	(0)	(1) 2.0%	(33) 64.7%
Mixed distressed (*n* = 149)	(29) 19.5%	(7) 4.7%	(2) 1.3%	(111) 74.5%

The results were computed based on group sizes

## Discussion

### Psychological and somatic distress in Chinese general hospital outpatients

Since questionnaires like PHQ-15/PHQ-9/GAD-7 could only provide a one-dimensional assessment of the somatic or psychological distress, the two dimensional assessment and four distress patterns provided a more complete picture. Our study confirmed that psychological burden was not rare in the Chinese general hospital outpatients, especially among those with high somatic distress. According to Lee’s study, 5.0, 15.8, and 10.0% of the general population in Hong Kong have been identified as having somatic, psychological, and mixed distress, respectively [17]. Due to our study design, the proportion of each distress pattern could not stand for its distribution in the whole sample of Chinese general hospital outpatients. Nevertheless, our results showed that patients with mixed distress and somatic distress alone were much common. The distribution of distress patterns between mainland and Hong Kong Chinese might be different. For example, mainland Chinese could be more conservative in expressing their emotions, especially the elderly, and female patients, or patients with higher family income. Further study conducted in the general population of mainland Chinese will help to clarify. Still, this could be enlightening for Chinese clinicians that the demand for mental health service was high, even though psychosomatic medicine as part of a patient’s health care is only beginning in China [51].

### Somatic/psychological distress and psycho-behavioral characteristics

As evidenced by lower health anxiety and higher SOC, our study showed that patients with mixed distress were strained to the greatest extent in comprehending and managing their illnesses. Since health anxiety has been found to be closely associated with somatization in both Western [27] and Chinese populations [28], and SOC apparently is a resource that enabled people to comprehend, manage and find meaning in their suffering, we have expected that somatically distressed patients had a higher level of health anxiety and lower level of SOC. On the contrary, our study found that psychologically distressed patients had a comparable level of heath anxiety as their somatically distressed counterparts, and a significantly lower level of SOC. Possible explanation could be that previously surveyed patients with somatoform disorders were more likely to resemble the mixed distressed patients identified in our study, instead of those with somatic distress alone. And psychological distress itself could also influence patients’ cognition negatively. Similarly, a systematic review found that the SOC was strongly related to and predictive of perceived health, especially mental health [52]. Our previous research also found that SOC was correlated with somatic symptom severity as well as the level of depression and generalized anxiety, with stronger correlation coefficients being found for depression and anxiety [44].

In addition, our study revealed that patients with psychological distress visited doctors less frequently, despite their high level of health anxiety and difficulties in understanding their condition. Lee’s community-based study also found that only somatic distress predicted health service utilization [17]. As the author noted, the potential reason could be that Chinese people tend not to view psychosocial complaints as a disorder and, thus, prefer to use self-help methods instead of seeking professional help. In addition, it is important to notice that mixed distress patients visited doctors more frequently. Therefore, somatic symptoms might have served as opportunities or “ticket behavior” for them to visit a doctor [53].

In terms of their doctor–patient relationship, psychologically distressed patients and their doctors rated their DPR as more difficult than those with only somatic distress. For a long time, it has been believed that patients with medically unexplained symptoms or somatoform disorders tended to be more unsatisfied with their doctor. As discussed above, it is probably that patients with somatoform disorders addressed mixed distress in our study, instead of somatic distress only. However, it is important to note that psychological distress might play a more important role in DPR than we previously hypothesized. As a previous study suggested, rather than somatic symptom severity, it is the level of depression that predicted patients’ experiences of the DPR [54]. Therefore, we assume that multiple somatic symptoms could have an indirect relationship with patients’ experiences of the DPR via psychological problems. However, our cross-sectional study design and exploratory research into this topic could not provide solid evidence. To better understand the interrelationships, a new data set from the longitudinal research is needed to test the model with mediation analysis.

Besides, our discriminate analysis and the classification results showed that large percentages of somatically distressed and psychologically distressed patients were misclassified. This furthered reminded us that, in terms of psycho-behavioral characteristic, psychologically distressed individuals resembled those with mixed distress. However, such classification prediction was based on the group sizes. Further studies with different samples and possibly different distribution of distress patterns are needed to examine the predictive ability of those demographic and psycho-behavioral characteristics.

Our study has several limitations. First, an equal number of participants with high and low somatic distress were recruited according to the study design, so that the proportions of different distress patterns in our study could not illustrate their distribution among Chinese general hospital outpatients. In addition, the sample only included patients from the internal medicine. Characteristics of patients from surgical and other departments remained unknown. Furthermore, to ensure the reliability of our results, patients with cognitive impairment, organic brain disorder, dementia, psychosis, and acute suicidal tendency were excluded, which could decrease the proportion of psychological distress. Second, the study was based on general hospital outpatients instead of a community population. Therefore, we did not know the characteristics of somatically or psychologically distressed patients who did not consult with a doctor. Third, only self-report questionnaires have been used to measure somatic and psychological distresses. Structured interviews and psychiatric diagnoses are needed in the future to confirm the spectrum of mental disorders of Chinese outpatients in general hospitals. Fourth, the Chinese versions of the SOC-9, PDRQ-9, and DDPRQ-10 have not been validated, even though their reliability and validity were found to be satisfactory in our study, as mentioned in the methods section. Moreover, even though the measurements have predominantly originated under Western cultural contexts. Future researches could explore the most common emotional and physical complaints of Chinese to better reflect their distress patterns.

## Conclusions

In conclusion, our study found that patients with psychological burden were not rare in the Chinese general hospital outpatients, who, however, were less likely to seek help despite the severe psychosocial impairment unless they were bothered by somatic distress at the same time. Therefore, the demand and challenge for mental health service in China were high. Gender, health anxiety, sense of coherence, mental quality of life, and frequent doctor visits could help to identify different distress patterns.

## Abbreviations

DDPRQ-10: the difficult doctor–patient relationship questionnaire
DPR: doctor–patient relationship
GAD-7: the seven-item anxiety scale
PDRQ-9: the patient–doctor relationship questionnaire
PHQ-9: the nine-item depression scale of the patient health questionnaire
PHQ-15: the 15-item somatic symptom scale of the patient health questionnaire
QoL: quality of life
SF-12 MCS: mental composite score of the 12-item short-form health survey
SF-12 PCS: physical composite score of the SF-12
SOC: sense of coherence
SOC-9: the nine-item sense of coherence scale
TCM: Traditional Chinese Medicine
WI-7: the seven-item Whiteley Index
