# Correction to: Psychological and somatic distress in Chinese outpatients at general hospitals: a cross-sectional study

**DOI:** 10.1186/s12991-018-0177-3

**Published:** 2018-02-05

**Authors:** Nana Xiong, Jing Wei, Kurt Fritzsche, Rainer Leonhart, Xia Hong, Tao Li, Jing Jiang, Liming Zhu, Guoqing Tian, Xudong Zhao, Lan Zhang, Rainer Schaefert

**Affiliations:** 1 Department of Psychological Medicine, Peking Union Medical College Hospital, Chinese Academy of Medical Sciences & Peking Union Medical College, Beijing, 100730 People’s Republic of China; 2 Department of Psychosomatic Medicine and Psychotherapy, University Medical Centre Freiburg, Freiburg, Germany; 3 Institute of Psychology, University of Freiburg, Freiburg, Germany; 4 Department of Gastroenterology, Peking Union Medical College Hospital, Chinese Academy of Medical Sciences & Peking Union Medical College, Beijing, China; 5 Department of Traditional Chinese Medicine, Peking Union Medical College Hospital, Chinese Academy of Medical Sciences & Peking Union Medical College, Beijing, China; 6 Department of Psychosomatic Medicine, Dongfang Hospital, School of Medicine, Tongji University, Shanghai, China; 7 Mental Health Centre, West China Hospital, Sichuan University, Chengdu, Sichuan China; 8 Department of Psychosomatics, Medical Division, University Hospital Basel, Basel, Switzerland

## Correction to: Ann Gen Psychiatry (2017) 16:35 10.1186/s12991-017-0158-y

Unfortunately, after publication of this article [1], it was noticed that the affiliation of Rainer Schaefert is incorrectly displayed as, “Department of General Internal Medicine and Psychosomatics, University Hospital Heidelberg, Heidelberg, Germany”. The correct affiliation should read, “Department of Psychosomatics, Medical Division, University Hospital Basel, Basel, Switzerland” and can be seen in the author details of this correction.
